# Type 3 Secretion System Cluster 3 Is a Critical Virulence Determinant for Lung-Specific Melioidosis

**DOI:** 10.1371/journal.pntd.0003441

**Published:** 2015-01-08

**Authors:** Maria G. Gutierrez, Tia L. Pfeffer, Jonathan M. Warawa

**Affiliations:** 1 Department of Microbiology and Immunology, University of Louisville, Louisville, Kentucky, United States of America; 2 Center for Predictive Medicine, University of Louisville, Louisville, Kentucky, United States of America; University of Tennessee, United States of America

## Abstract

*Burkholderia pseudomallei*, the bacterial agent of melioidosis, causes disease through inhalation of infectious particles, and is classified as a Tier 1 Select Agent. Optical diagnostic imaging has demonstrated that murine respiratory disease models are subject to significant upper respiratory tract (URT) colonization. Because human melioidosis is not associated with URT colonization as a prominent presentation, we hypothesized that lung-specific delivery of *B. pseudomallei* may enhance our ability to study respiratory melioidosis in mice. We compared intranasal and intubation-mediated intratracheal (IMIT) instillation of bacteria and found that the absence of URT colonization correlates with an increased bacterial pneumonia and systemic disease progression. Comparison of the LD_50_ of luminescent *B. pseudomallei* strain, JW280, in intranasal and IMIT challenges of albino C57BL/6J mice identified a significant decrease in the LD_50_ using IMIT. We subsequently examined the LD_50_ of both capsular polysaccharide and Type 3 Secretion System cluster 3 (T3SS3) mutants by IMIT challenge of mice and found that the capsule mutant was attenuated 6.8 fold, while the T3SS3 mutant was attenuated 290 fold, demonstrating that T3SS3 is critical to respiratory melioidosis. Our previously reported intranasal challenge studies, which involve significant URT colonization, did not identify a dissemination defect for capsule mutants; however, we now report that capsule mutants exhibit significantly reduced dissemination from the lung following lung-specific instillation, suggesting that capsule mutants are competent to spread from the URT, but not the lung. We also report that a T3SS3 mutant is defective for dissemination following lung-specific delivery, and also exhibits *in vivo* growth defects in the lung. These findings highlight the T3SS3 as a critical virulence system for respiratory melioidosis, not only in the lung, but also for subsequent spread beyond the lung using a model system uniquely capable to characterize the fate of lung-delivered pathogen.

## Author Summary

Respiratory melioidosis is a lethal disease presentation of the bacterium *Burkholderia pseudomallei*, which is found in tropical regions worldwide. Respiratory melioidosis has also been highlighted as a concern in the biodefense community given the potential for weaponization of *B. pseudomallei*. This study demonstrates that respiratory melioidosis models can significantly vary in their disease presentations in mice, depending on whether the upper respiratory tract represents an initial site of infection. We have demonstrated that lung-specific infections of mice, which avoid nasal cavity colonization, result in a course of disease with greater maturation of pneumonia and systemic spread, and we propose that this represents a critical advance in the field of studying respiratory melioidosis. We further characterize that the capsule virulence determinant, previously considered important for respiratory melioidosis, has reduced significance when characterized in the context of lung-specific disease, while the Type 3 Secretion System cluster 3 is a critical virulence determinant for *B. pseudomallei* required for efficient colonization of the lung as well as spread to other tissues.

## Introduction

*Burkholderia pseudomallei* is the Tier 1 Select Agent bacterial pathogen responsible for the disease melioidosis. *B. pseudomallei* is found in moist tropical soils worldwide, but has been long characterized to be endemic to Southeast Asia and northern Australia [1]. Naturally acquired disease typically involves percutaneous inoculation or inhalation of pathogen by susceptible hosts, with risk factors including diabetes and alcoholism [2]. Under exceptional conditions, such as natural disasters, otherwise healthy individuals are also susceptible to melioidosis [3], [4], [5], [6], suggesting that dose and route of inoculation are key elements to determining whether or not a healthy individual acquires disease. The ability of *B. pseudomallei* to establish a lethal respiratory disease, combined with its inherent resistance to numerous classes of antibiotics, highlights the importance of characterizing respiratory melioidosis for the purposes of biodefense. Importantly, no licensed vaccine exists for melioidosis, nor for glanders, which is caused by the very closely related pathogen *Burkholderia mallei*.

Respiratory melioidosis has been well studied in surrogate animal models for both basic science investigations as well as therapeutic studies. *B. pseudomallei* has a significant lung tropism, irrespective of the route of acquisition [2], and is able to spread to other tissues to cause a lethal systemic disease. Bioluminescent *B. pseudomallei* strains have been generated which allow for the temporal assessment of disease progression in individual animals using optical diagnostic imaging. In the first of such studies, a non-lethal intranasal challenge revealed that *B. pseudomallei* prominently colonizes the upper respiratory tract (URT) of mice, leading to a rapid development of meningitis within 24 hr, likely resulting from spread to the olfactory bulbs via olfactory nerve endings [7]. Interestingly, the symptoms of a prominent URT colonization (rhinitis, sinusitis, tonsillitis, laryngitis, and otitis media) have not been described as common presentations of melioidosis [8], [9], suggesting that URT infections do not play the prominent role in humans as the one observed in murine models. Indeed, a large clinical sampling study of throat swabs revealed no carriage of *B. pseudomallei* in healthy volunteers, while melioidosis patients had culturable *B. pseudomallei* from the throat in 36.1% of cases [10] – less than the presentation rate of pneumonia at 50% [11]. Additionally, paired analyses of throat and sputum carriage from the same patient demonstrated that *B. pseudomallei* culture from the throat is underrepresented relative to sputum culture, suggesting that presence of *B. pseudomallei* in the lung is not a direct result of a descending infection from a colonized throat [10]. These clinical trends, combined with the relatively rare presentation of meningitis at an incident rate of 4–5% [11], [12], suggest that murine models in which *B. pseudomallei* is delivered intranasally over-represent the incidence of URT disease and CNS involvement in studies of respiratory melioidosis. Significant URT colonization was also observed by diagnostic optical imaging in a lethal intranasal murine model of respiratory melioidosis [13], suggesting that the URT colonization phenotype is not specific to sub-acute disease.

The role of murine URT colonization on studies of respiratory melioidosis is poorly understood, though may have a significant impact on both basic and translational studies. A recent study investigating an intratracheal instillation of *B. pseudomallei* directly into the lungs successfully demonstrated that avoidance of initial URT colonization could limit both CNS involvement and late stage URT colonization [14]. This finding is consistent with other studies demonstrating the mechanism by which melioidosis-associated meningitis arises from spread to the brain from the nasal cavity within 24 hr using both olfactory and trigeminal nerves [15]. Because intratracheal delivery is capable of avoiding such URT and CNS involvement, an unanswered question is what impact these cephalic disease presentations have on disease outcome of pneumonic and systemic disease. We have improved upon other published non-surgical approaches to deliver bacteria directly into the lung as a novel instillation strategy termed intubation-mediated intratracheal (IMIT) inoculation, which we have validated to provide >98% efficiency in pulmonary delivery [16], and we therefore used this highly accurate lung inoculation approach to study the impact of lung-specific melioidosis on dissemination and disease outcome. Additionally, we investigated whether lung-specific administration of capsular polysaccharide and Type 3 Secretion mutants exhibit modified courses of disease relative to previous characterization in other respiratory murine models. Using a combination of optical diagnostic imaging, targeted lung-specific delivery of *B. pseudomallei*, and previously characterized virulence system mutants, we demonstrate that the presence or absence of URT infection in murine models exhibits significant disease outcome differences, with potential impacts on both basic and translational studies.

## Methods

### Bacterial strains and media

*Burkholderia pseudomallei* strains were routinely cultured in Lennox Broth (LB) at 37°C. In preparation for infection studies, *B. pseudomallei* strains were subcultured 1∶25 from overnight LB cultures into dialyzed and chelated Trypticase Soy Broth (TSBDC [17]) supplemented with 50 µM monosodium glutamate and grown for 3 hr at 37°C with shaking. Antibiotics were used at the following concentrations: kanamycin, 25 µg/ml; polymyxin B, 50 µg/ml; and streptomycin, 100 µg/ml.

### Luminescent *B. pseudomallei*

Luminescent *B. pseudomallei* strains JW280 and JW280 Δ*wcb* were generated and described elsewhere [13]. JW280 Δ*sctU*_Bp3_ was generated by allelic exchange by using the S17-1/pKAS46-araP_tolC_ *lux* construct [13] to add the *luxCDABE* operon to DD503 Δ*sctU*_Bp3_ [18], as previously described.

### Ethics statement

These studies were approved by the University of Louisville Institutional Animal Care and Use Committee (Protocol numbers 10073 and 13053) in agreement with NIH guidelines and the “Guide for the Care and Use of Laboratory Animals” (NRC). In-house breeding of mice was conducted on Protocol 11113.

### Mouse respiratory infections

Animal studies were conducted under Biosafety Level 3 conditions using eight to ten-week-old female or male albino C57BL/6J mice (B6(Cg)-Tyr^c-2J^/J, Jackson Laboratories and in-house breeding). Freshly grown bacteria were washed into phosphate buffered saline (PBS) to appropriate concentrations for infection using OD_600_-based calculations. Intranasal infections were carried out as previously described using 30 µl *B. pseudomallei* suspensions [19]. Intubation-mediated intratracheal (IMIT) instillation was performed, as previously described, to facilitate non-surgical lung-specific disease [16], [20]. Briefly, mice were isoflurane-anesthetized and 2% lidocaine was applied to the back of the mouth as a local anesthetic. Mice were intubated using a 20 gauge catheter, and catheter placement was confirmed by flow meter. Bacterial suspensions were directly instilled into the lung using a 22 gauge blunt needle inserted through the catheter. Optical diagnostic imaging was conducted with an IVIS Spectrum (Caliper Life Sciences) as described previously [13], [21], with once to twice-daily imaging through day 5, and once daily thereafter until the study completion at day 14. Animals were euthanized at the onset of moribund disease which was defined by loss of righting reflex.

### Bacterial enumeration at key sites of infection

Infected animals were euthanized and necropsied to enumerate bacteria from infected tissues. Blood samples were collected by cardiac puncture, while bronchoalveolar lavage (BAL) was collected in 1 ml of PBS. Necropsied tissues were subjected to bioluminescence imaging (IVIS Spectrum) in a 24 well black plate before serial dilution enumeration of bacterial burden from tissue homogenate, as described elsewhere [21]. We have defined correlations between tissue cps and CFU specifically for each tissue as follows: Lung, log(cps) = 1.219log(CFU) – 2.999 (R^2^ = 0.68); Liver, log(cps) = 1.238log(CFU) – 3.502 (R^2^ = 0.99); Spleen, log(cps) = 1.088log(CFU) – 1.741 (R^2^ = 0.98).

### Statistical analyses

Student T-test, one-way ANOVA, two-way ANOVA and survival analyses (Mantel-Cox test and Gehan-Breslow-Wilcoxon test) were conducted in GraphPad Prism. Probit analysis (Finney Method, StatPlus 2009 Professional) was used to calculate LD_50_ +/− standard error, which was subsequently subjected to Student T-test analysis to investigate significant differences of LD_50_ values of different *B. pseudomallei* strains.

## Results

### Lung-specific delivery of *B. pseudomallei* impacts disease maturation

**Figure 1:**
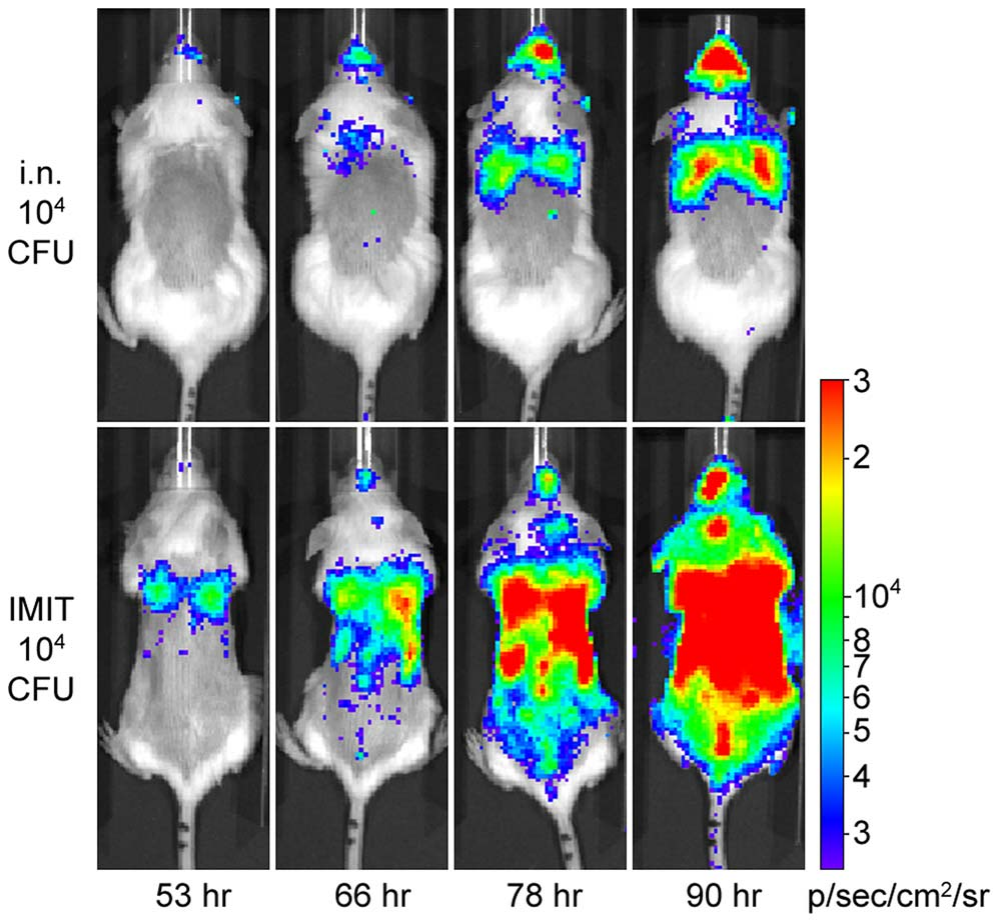
*In vivo* optical diagnostic imaging of respiratory melioidosis. Female albino C57BL/6J mice were challenged with 10^4^ CFU of luminescent *B. pseudomallei* strain JW280 by either the intranasal (Top panel) or IMIT (Bottom panel) routes of infection. Mice were imaged twice daily beginning at 18 h post infection. Images were uniformly adjusted to a range of 2.5×10^3^ to 3×10^4^ p/s/cm^2^/sr on a logarithmic scale. A representative panel of images beginning 53 hr post infection is presented for both infection routes, where the last image of each panel represents the moribund disease endpoint.

Opportunistic URT colonization by *B. pseudomallei* is associated with high rates of meningitis in murine respiratory models [7], [22], and avoidance of bacterial deposition on the nasal mucosa by intratracheal instillation is associated with reduced CNS involvement [14]. We decided to investigate whether the URT colonization typical of current respiratory melioidosis models impacts the moribund disease presentation. To investigate this, we employed a non-surgical approach that would facilitate direct instillation of bacteria directly into the lungs of mice with >98% efficacy [16], termed intubation-mediated intratracheal (IMIT) delivery [20]. Albino C57BL/6J mice were used as a model system in these studies given the myriad of transgenic tools available in the C57BL/6J background, and the importance of coat color in optimizing detection of bioluminescent bacterial pathogens [21]. Thus, albino C57BL/6J mice were infected with 10^4^ CFU of luminescent *B. pseudomallei* strain, JW280, using either intranasal or IMIT delivery and monitored twice daily by optical diagnostic imaging. While challenge with 10^4^ CFU by both routes of inoculation resulted in moribund disease over a similar time frame, the foci of disease in moribund animals was dramatically different (Fig. 1). As observed in our previous work with a BALB/c model [13], C57BL/6J mice infected intranasally developed a significant URT infection with a reduced bioluminescent signal associated with the thoracic cavity/lung (Fig. 1). Interestingly, mice infected in a lung-specific manner by IMIT developed a pulmonary infection earlier than by intranasal delivery of the same inoculum, consistent with estimates that 10% of an intranasal inoculum is delivered to the lung for other respiratory pathogens [23]. Importantly, the early involvement of the lung in the IMIT model led to a more mature pneumonia than that observed in the intranasal model, with subsequent systemic spread not previously observed in the intranasal model. This suggests that mice are capable of sustaining an advanced systemic disease than previously thought possible in the intranasal model, and therefore that URT colonization by *B. pseudomallei* directly contributes to the host morbidity of the intranasal model. These data indicate that avoiding initial deposition of bacteria in the upper respiratory mucosa represents an important modification for studying mature pneumonia development and subsequent systemic spread.

### Respiratory melioidosis models exhibit similar disease kinetics

**Figure 2:**
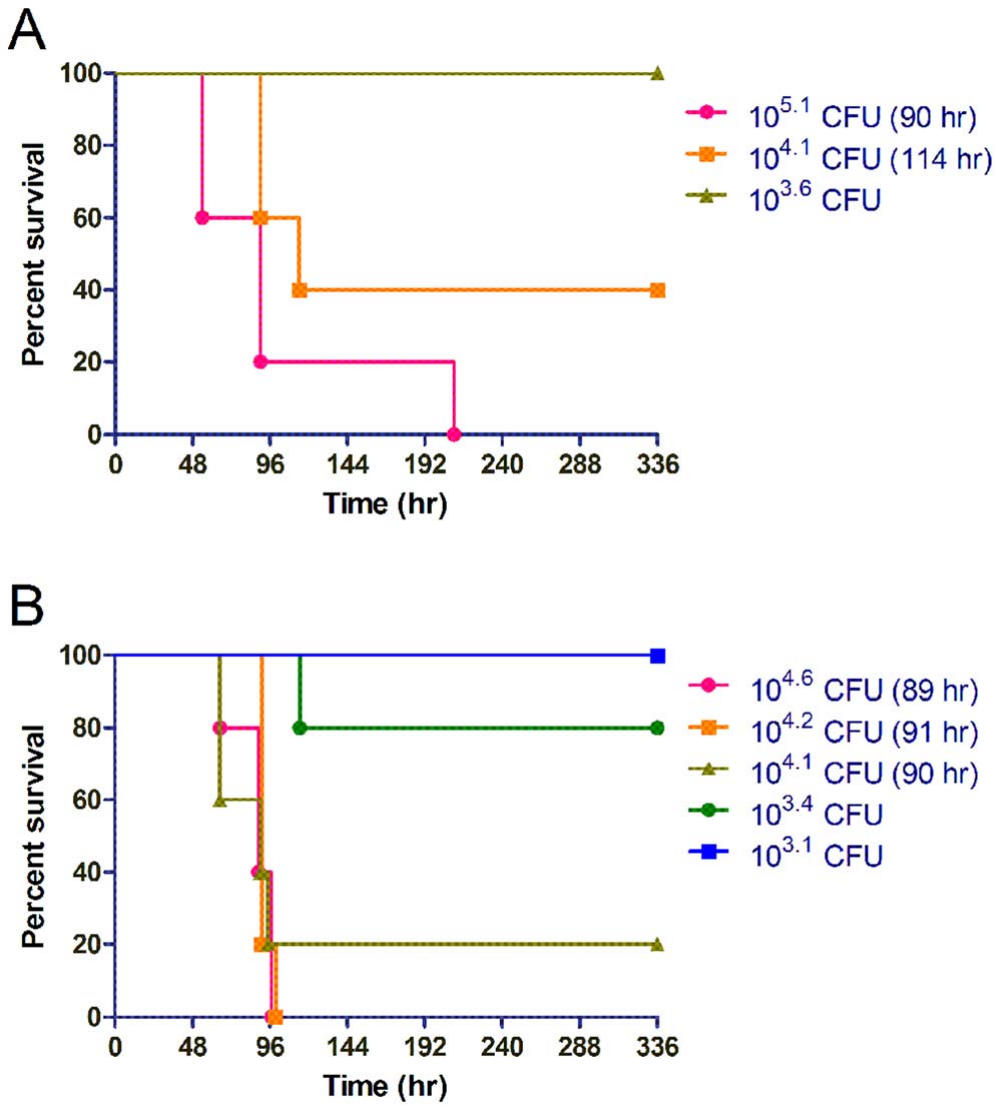
Host response to lung-specific delivery of *B. pseudomallei*. Groups of 5 female albino C57BL/6J mice were challenged with increasing doses of luminescent *B. pseudomallei* strain JW280 by either the intranasal (A) or IMIT (B) routes of infection. Mice were monitored for 14 days (336 hr) for disease progression and euthanized at the onset of moribund disease presentation. Survival curves are used to present the dose-dependent response of the host to increasing bacterial challenges. The MTTD was calculated for groups with ≥50% mortality, as indicated.

We decided to examine whether the kinetics of respiratory disease vary as a function of the method of delivery in order to investigate whether the involvement of different foci of infection impacts the median time to death (MTTD). Mice were infected by either intranasal or IMIT instillation and both respiratory disease models were able to establish an acute course of disease in C57BL/6J mice with a typical MTTD of ∼4 days (Fig. 2). We observed typical dose response disease susceptibility in the intranasal model with a transition from 100% survivors to 100% fatality over a 32 fold dose range (Fig. 2A). Interestingly, we observed a much sharper dose transition in host susceptibility to disease in the IMIT-infected groups, which occurred over a single ten-fold dose range (Fig. 2B). We calculated the LD_50_ of the intranasal C57BL/6J model to be 12.1±2.4×10^3^ CFU, while the IMIT model significantly lowered the LD_50_ to 5.4±2.0×10^3^ CFU (P = 0.04). Thus, targeting of *B. pseudomallei* directly into the lungs of mice resulted in a lowering of the LD_50_ and resolved host susceptibility into a more discrete dose range transition from host susceptibility to clearance of pathogen. Importantly, the small 2.2 fold change in the LD_50_ and similar MTTD at equivalent doses suggest that the ultimate course of disease takes place over a similar time frame, regardless of the method of inoculation, but that the disease presentation is dramatically altered dependent on whether *B. pseudomallei* spreads specifically from the lung, or whether initial inoculation prominently colonizes the URT. We conclude that intranasal and IMIT respiratory infections therefore cause very different morbidity in the host as a result of either primarily URT or systemic endpoints, respectively.

**Figure 3:**
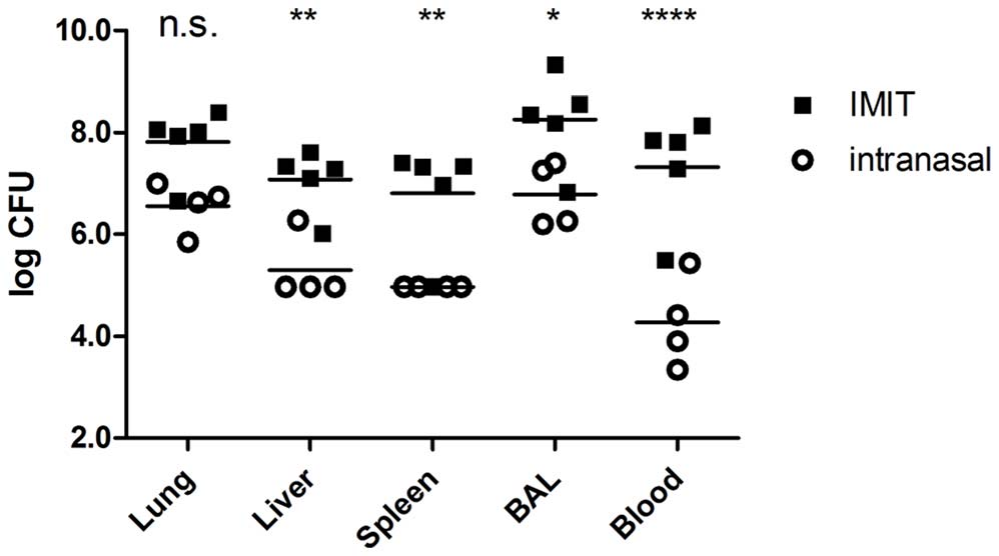
Bacterial enumeration at moribund disease for respiratory melioidosis models. Groups of 5 female albino C57BL/6J mice were infected by either the i.n. (10^5.1^ CFU) or IMIT (10^4.6^ CFU) routes of infection and euthanized at moribund disease endpoints. Bacteria were enumerated from tissues homogenized in 1 ml PBS, from a 1 ml PBS BAL collection, or from cardiac-drawn blood. Bacterial burden was calculated as CFU/tissue (lung, liver, and spleen) or bacteria per ml of body fluid (BAL and blood). Significant differences between log transformed data were evaluated by 2-way ANOVA with Bonferroni multiple comparisons (n.s., not significant; *, p<0.05; **, p<0.01; ****, p<0.0001).

To further investigate the role of URT colonization on disease endpoints, we subsequently investigated the bacterial burdens of tissues isolated from moribund mice to further characterize differences in bacterial dissemination. Tissues were necropsied from moribund mice infected with ∼LD_100_ doses of JW280 by either the intranasal or IMIT routes of infection, and we compared the tissue burdens of moribund animals for the lung, liver, spleen, BAL and blood of mice infected with (i.n.) or without (IMIT) involvement of the URT. Consistent with the findings of Fig. 1, we found that IMIT delivery of *B. pseudomallei* facilities significant disease maturation in all monitored tissues, both at the primary site of infection in the lung, as well as in the disseminated infection of the liver and spleen (Fig. 3). Further, we observe a >3 log CFU difference of bacterial dissemination through the blood, indicating that moribund mice exhibit a greater degree of septicemia in the IMIT model relative to the i.n. model. Consistent with *in vivo* diagnostic imaging, bacterial tissue burden analysis reveals that *B. pseudomallei* infections involving prominent URT colonization result in host morbidity associated with reduced disease maturation in core body sites, suggesting that the host morbidity of the i.n. model is directly influenced by the bacterial colonization of the nasal cavity.

### Sex difference influence host susceptibility to lung-specific melioidosis

**Figure 4:**
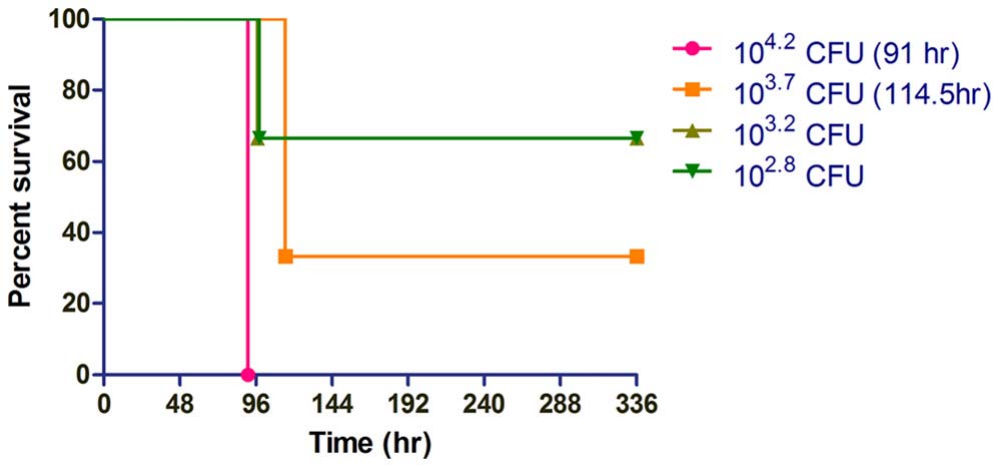
Male host response to lung-specific delivery of *B. pseudomallei*. Groups of 3 male albino C57BL/6J mice were challenged with increasing doses of luminescent *B. pseudomallei* strain JW280 by IMIT infection. Mice were monitored for 14 days (336 hr) for disease progression and euthanized at the onset of moribund disease presentation. Survival curves are used to present the dose-dependent response of the host to increasing bacterial challenges. The MTTD was calculated for groups with ≥50% mortality, as indicated.

Given a recent focus in the scientific community on understanding disease progression in both male and female model systems [24], we additionally performed survival analyses in male albino C57BL/6J mice to investigate whether sex differences impact susceptibility to lung-specific respiratory melioidosis. Male disease progression closely mirrored that observed in the female models (Fig. 2 and 4), where in both cases, the 100% minimally lethal dose was observed at 10^4.2^ CFU by IMIT, with a 91 hr MTTD. We calculated the LD_50_ for lung-specific melioidosis in male mice as 1.9±1.2×10^3^ CFU, which was 2.9 fold reduced relative to the female LD_50_ (P = 0.25). Thus, male mice are not significantly different in their susceptibility to respiratory melioidosis relative to female mice in the C57BL/6J IMIT model system.

### Type 3 Secretion is more critical for respiratory melioidosis than capsule

**Figure 5:**
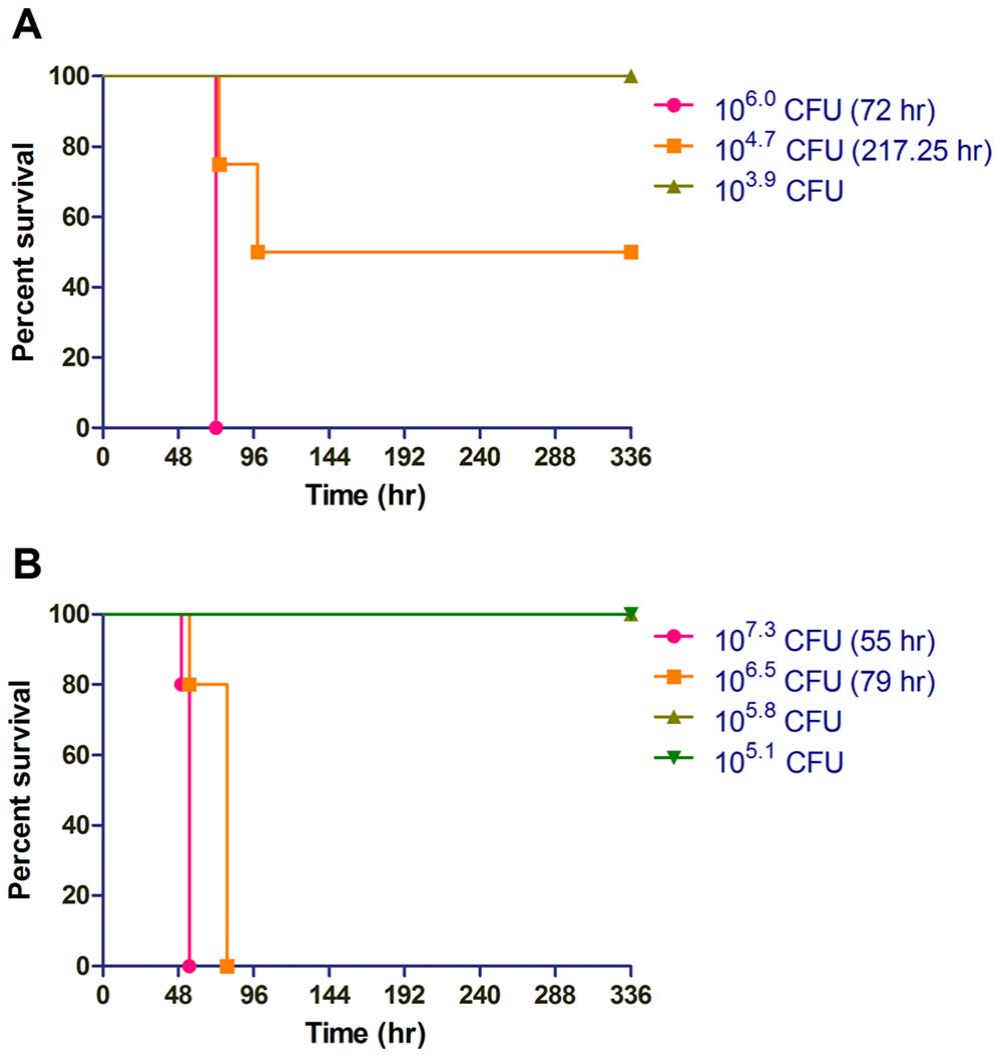
Host response to capsule and T3SS3 mutants of *B. pseudomallei*. Groups of 5 female albino C57BL/6J mice were challenged by the IMIT route of infection with increasing doses of either Δ*wcb* capsule mutant (A) or Δ*sctU*_Bp3_ T3SS3 mutant (B) in the luminescent *B. pseudomallei* strain JW280 background. Mice were monitored for 14 days (336 hr) for disease progression and euthanized at the onset of moribund disease presentation. Survival curves are used to present the dose-dependent response of the host to increasing bacterial challenges of *B. pseudomallei* mutants. The MTTD was calculated for groups with ≥50% mortality, as indicated.

We observed that URT colonization impacts disease outcome in the murine respiratory melioidosis model, and we therefore hypothesized that URT colonization could impact our basic understanding of the role of virulence determinants in mediating *B. pseudomallei* pathogenesis. Previous murine studies demonstrated that a capsule mutant LD_50_ is attenuated 10^1.8^ fold in a respiratory melioidosis model [19], and that T3SS3 is also required for the full virulence in an equivalent dose challenge [25]. We therefore examined the response of albino C57BL/6J mice infected with increasing doses of either a luminescent capsular polysaccharide mutant (JW280 Δ*wcb*) or a T3SS3 mutant (JW280 Δ*sctU*_Bp3_). We found that a capsule mutant inoculated by IMIT was not significantly attenuated relative to the wild type strain with a capsule mutant LD_50_ calculated to be 10^4.57^ CFU (6.8 fold attenuation, P = 0.60). We observed a MTTD of 72 hr at a ∼LD_100_ challenge (Fig. 5a), which represents a faster course of disease than the a ∼LD_100_ dose of wild type at 91 hr, albeit with a larger challenge dose. Thus, the JW270 capsular polysaccharide mutant is not significantly attenuated in the lung-specific IMIT model, contrasting with our prior findings in the i.n. model. In the IMIT model, the T3SS3 mutant had a calculated LD_50_ of 10^6.19^ CFU, which represents a significant attenuation of 10^2.5^ fold (P = 0.004). The course of disease of the T3SS3 was observed to have a MTTD of 79 hr at the minimally lethal dose (Fig. 5b), which like the capsule mutant strain was faster than the wild type MTTD, albeit with a larger challenge dose. Thus, in a lung-specific respiratory melioidosis model, T3SS3 is a critical virulence determinant for *B. pseudomallei* in the lung, whereas the capsular polysaccharide appears to play a more minor role.

### Capsule and T3SS3 mutants are defective in spread from the lung

**Figure 6:**
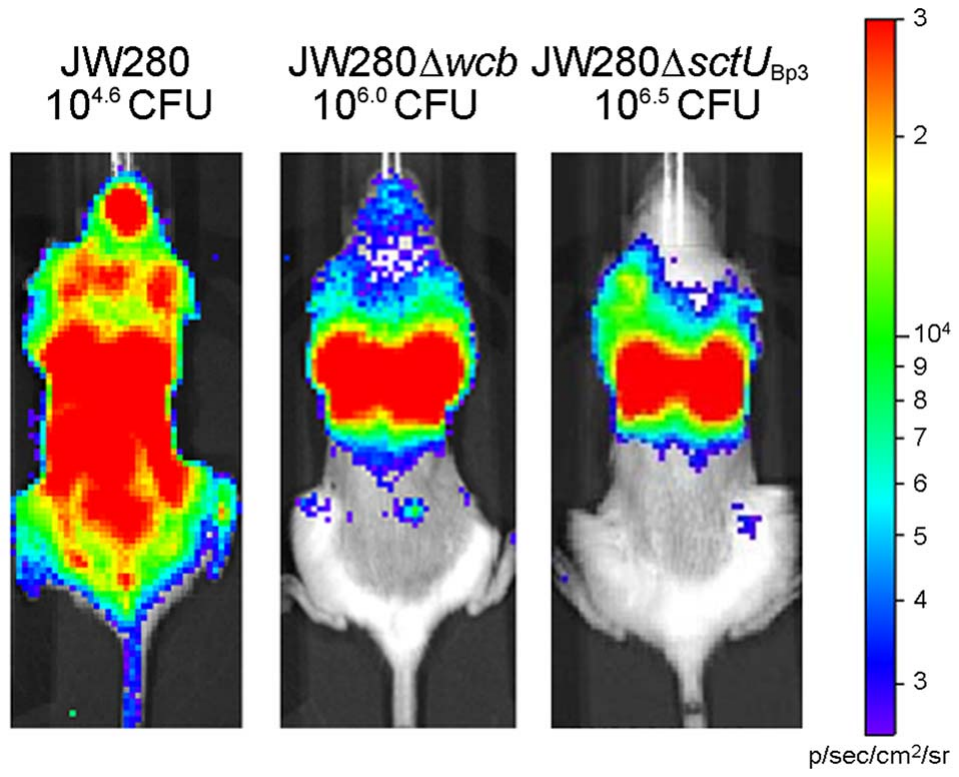
Detection of dissemination of *B. pseudomallei* mutants by optical imaging. Groups of 5 female albino C57BL/6J mice were challenged by the IMIT route of infection with either wild type luminescent *B. pseudomallei* strain JW280, the Δ*wcb* capsule mutant, or the Δ*sctU*_Bp3_ T3SS3 mutant. Representative images of disease endpoints are presented, with uniform image settings adjusted to a range of 2.5×10^3^ to 3×10^4^ p/s/cm2/sr on a logarithmic scale.

**Figure 7:**
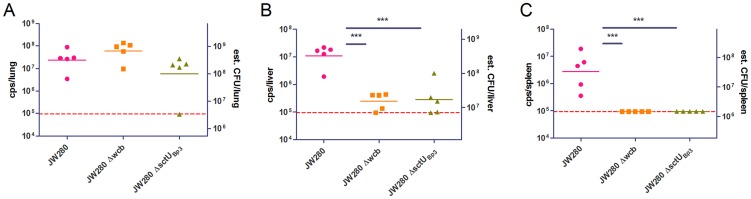
Bacterial enumeration of *B. pseudomallei* mutants in moribund respiratory disease. Groups of 5 female albino C57BL/6J mice were infected with wild type JW280 (10^4.2^ CFU), Δ*wcb* capsule mutant (10^6.0^ CFU), or Δ*sctU*Bp3 T3SS3 mutant (10^6.5^ CFU), and euthanized at moribund disease endpoints. Bacteria were enumerated from tissues *ex vivo* by optical imaging (left Y-axis: cps/tissue) with presentation of the estimated tissue CFU burdens based on calculated tissue-specific cps:CFU correlation (right Y-axis: CFU/tissue est.) for lung (A), liver (B) and spleen (C). The 95% LOD was calculated as a technical background luminescence and indicated as a dotted horizontal line. Data points below the 95% LOD were set to the 95% LOD value. Significant differences (1-way ANOVA with Tukey posttest) between log transformed data sets are indicated with an adjoining line (*, p<0.05; **, p<0.01; ***, p<0.001).

We decided to further investigate whether abrogation of URT colonization in our lung-specific disease studies impacts the dissemination potential of the capsule and T3SS3 mutants. We performed optical diagnostic imaging of ∼LD_100_ infections of the wild type strain as well as both the capsule and T3SS3 mutants to characterize bacterial burdens at moribund disease. We found that the wild type strain is capable of dissemination beyond the lung to colonize all sites of the body at high titer (Fig. 6). The capsule and T3SS3 mutants developed significant bacterial pneumonia yet exhibited a spread deficiency with minimal bacterial burden outside of the lung (Fig. 6). We further characterized the dissemination defects of these mutants by enumerating bacteria from the lung, liver and spleen, from mice infected with minimally lethal doses of each strain. We found that while all tested strains established bacterial pneumonias of 10^8^–10^9^ CFU per tissue, the capsule and T3SS3 mutants exhibited significant dissemination defects to the liver and spleen in both tissues (Fig. 7). These data demonstrate that a capsule mutant exhibits reduced fitness to disseminate from the lung, consistent with the previously characterized role of capsular polysaccharide in mediating complement protection [26]. Thus, a capsule mutant is capable of producing a lethal pneumonia with a similar LD_50_ as wild type, yet without the wild type-ability to spread beyond this organ. In contrast, the T3SS3 virulence determinant exhibits a significantly reduced fitness in the lung by LD_50_ analysis, and this reduced fitness is also associated with a reduced dissemination potential to the liver and spleen. Thus, both the capsule and T3SS3 mutants are spread deficient when delivered specifically to the lung, highlighting a major difference between the current lung-specific respiratory melioidosis model versus our previous work with i.n. models. This finding suggests that the differences in respiratory melioidosis models, with respect to URT involvement in dissemination and endpoint, dramatically influences our interpretation of basic science investigation of the role of *B. pseudomallei* virulence determinants.

### T3SS3 mutant exhibits reduced fitness in lung over the course of disease

**Figure 8:**
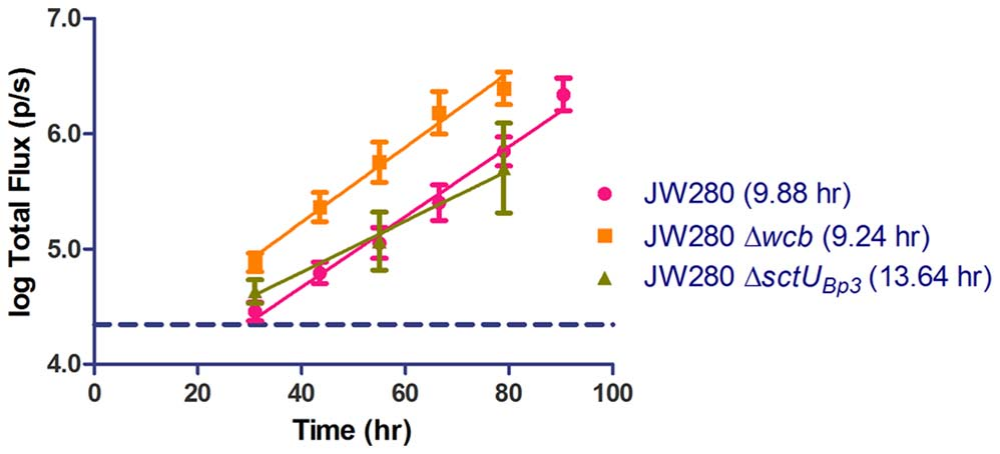
Detection of pulmonary growth rates of *B. pseudomallei* mutants *in vivo*. Groups of 5 female albino C57BL/6J mice were infected with wild type JW280 (10^4.2^ CFU), Δ*wcb* capsule mutant (10^6.0^ CFU), or Δ*sctU*Bp3 T3SS3 mutant (10^6.5^ CFU), and monitored by optical diagnostic imaging once to twice daily. ROIs from the dorsally-imaged thoracic cavity were plotted as a function of infection time for each mutant. The 95% LOD was calculated for the background luminescence of uninfected mice and indicated as a dotted horizontal line. The calculated doubling rate of bioluminescent signal of each strain is indicated.

We hypothesized that the reduced fitness characterized at moribund disease would similarly be associated with a reduced fitness throughout the course of disease. We therefore performed optical imaging of mice infected at a ∼LD_100_ dose of JW280, JW280 Δ*wcb*, or JW280 Δ*sctU*_Bp3_ and quantified the *in vivo* bioluminescence until moribund endpoints were reached. We identified an *in vivo* logarithmic increase in bioluminescence of all strains in the lung, but observed that the T3SS3 mutant exhibited a reduced fitness relative to both wild type and capsule mutant strains (Fig. 8). The bioluminescence doubling rate was calculated for all strains and we found non-significant differences of the doubling rates of the wild type and capsule mutant strains of 9.88 and 9.24 hr, respectively (One-way ANOVA/Tukey not significant). However, the T3SS3 mutant bioluminescence doubled at a significantly reduced rate of 13.64 hr (P<0.001), suggesting that the T3SS3 mutant is less fit to grow in host niches and/or is subject to enhanced clearance by the host. This finding is consistent with our study data, highlighting T3SS3 as a critical virulence determinant for *B. pseudomallei* lung colonization whereas the capsular polysaccharide plays a lesser role in disease of the lung.

## Discussion

We have previously developed an optical diagnostic imaging model of intranasal respiratory melioidosis and observed that the URT of mice infected in this manner are subject to prominent infection [13]. URT colonization is associated with infection of the nasal-associated lymphoid tissue (NALT) as well as infection of the olfactory bulbs/CNS [7], [15]. As discussed above, descriptions of disease states associated with URT infections have not been described in human melioidosis, and paired analysis of cultures sputum and throat swabs suggests that pneumonia gives rise to presence of *B. pseudomallei* at the top of the respiratory tract rather than URT carriage seeding a primary infection which descends to the lung [10]. The over-representation of these symptoms in mice have led us, and others, to investigate alternatives to the standard approaches of inoculating mice with *B. pseudomallei* through the nares. A recently developed intratracheal model of respiratory melioidosis succeeded in abrogating CNS infections, suggesting that URT colonization is directly responsible for the high levels of meningitis reported in the murine model [14], [27]. Our current studies focused on advancing these findings by identifying whether URT infection in the mouse impacts the overall course of disease and ask whether these impacts might influence both basic and translational studies of respiratory melioidosis.

Importantly, we found that inoculation of *B. pseudomallei* directly into the lung dramatically altered disease outcome, where we observed significant increases in both lung burden and septicemic spread not previously observed in intranasal inoculation studies. Our survival analysis of i.n. and IMIT-infected mice revealed that both routes of infection supported a disease process with very similar timing of inoculation to moribund endpoint; however, the major difference between the models was the difference in which host tissues supported the dominant site(s) of infection. IMIT also lowered the LD_50_ relative to the i.n. model, and provided an earlier development of pneumonia which progressed to an advanced systemic disease. Intranasal infection exhibits a clear bias to nasal cavity colonization, and conversely the IMIT model achieves systemic disease at moribund endpoints. This difference in infection site at moribund disease strongly suggests that the causation of moribund presentation is very different in these models, with IMIT providing systemic, general organ failure disease, while the moribund disease of the intranasal model is very directly related to the bacterial burden in the nasal cavity. Pathological analysis of the URT of mice infected by the i.n. route has revealed significant blockage by inflammatory cell debris in the nasal turbinates [27], thus the severe pathology/rhinitis of the intranasal model likely promotes moribund disease. Both the IMIT and i.n. models have moribund disease symptoms which include labored breathing of mice, and given that mice are obligate nasal breathers, we hypothesize that nasal cavity occlusion in the intranasal model drives moribund endpoints while the labored breathing of the IMIT model may reflect greater lung pathology, as the IMIT model supported >1 log more bacteria per lung than the i.n. model. Future studies will be required to investigate whether the aerosol model – which also would involve the nasal mucosa as a primary site of infection – is similarly is subject to preferential colonization of the URT over systemic spread.

The IMIT inoculation method we developed is distinct from other non-invasive intratracheal instillation methods, including those used previously for *B. pseudomallei* instillation [14]. IMIT inoculation is a two-step process in which mouse intubation is followed by instillation of bacteria via a long blunt needle, and the approach facilitates an intermediate confirmation of correct catheter placement into the trachea (rather than the esophagus), and is therefore not prone to user error associated with unintentional mis-inoculation of the GI tract [16]. IMIT inoculation also benefits from being a non-invasive approach which avoids overt deposition of bacteria into the blood stream which could occur as a result of surgical intratracheal inoculation.

This study incorporated use of albino C57BL/6J mice as a novel host model system in which to study respiratory melioidosis. A vast array of murine transgenic lines are available to the research community, the majority of which are available in the C57BL/6J background, which have been, and will continue to be important tools in melioidosis studies. C57BL/6 mice are commonly referred to as representing a chronic model of melioidosis, however both in the intranasal and IMIT infection studies we found that C57BL/6J mice develop an acute disease with a MTTD of 3–4 days. While C57BL/6J mice do appear to have a higher resistance to respiratory melioidosis with intranasal LD_50_ values 1–3 logs higher than their BALB/c counterparts [28], we conclude that C57BL/6J mice successfully model an acute respiratory disease presentation. We further made use of tyrosinase-negative mice which have albino coats and therefore offer greater sensitivity over black coated mice to detect bioluminescent bacteria, which is necessary to monitor the early stages of disease progression *in vivo*.

We hypothesized that the improved ability to study disease maturation of respiratory melioidosis in the absence of URT colonization might influence the role of virulence determinants in mediating *B. pseudomallei* pathogenesis. Given our prior interest in studying the role of capsular polysaccharide in mediating *B. pseudomallei* dissemination from the lung, we investigated the role of a capsule mutant using IMIT inoculation. Unlike our previous work which identified an attenuation of the Δ*wcb* capsule mutant of 10^1.8^–10^2.3^ fold in intranasal models [13], [19], we found a non-significant attenuation of just 6.8 fold (10^0.8^) in the IMIT model, suggesting that the capsular polysaccharide is not absolutely critical for the initial stages of lung colonization. From our growing understanding of the difference between i.n. and IMIT models, we conclude that the capsule mutant is attenuated in its ability to colonize the nasal mucosa as the contributor to its greater attenuation in the i.n. model, and conversely that capsular polysaccharide is not as critical for disease in the lung. More importantly, we further characterized whether the capsule mutant is required for dissemination beyond the lung. In our previous studies, we had found that there was no significant defect in dissemination of the capsular polysaccharide mutant when studied at the minimally lethal dose in the murine intranasal model [19]. This previous observation had not been anticipated given the previous demonstration that capsular polysaccharide is required to resist opsonization by host complement likely during dissemination through the blood stream [26], and further that capsule is a critical virulence determinant in systemic disease models of both the hamster and mouse with an attenuation of ∼10^5^ fold [29], [30]. Importantly, our current studies provide a modified understanding of the role of capsular polysaccharide in mediating dissemination beyond the lung, as we now observe that a capsule mutant is defective in lung-specific dissemination both optical diagnostic imaging as well as tissue burden analysis. We retrospectively interpret our previous studies to suggest that capsular polysaccharide mutants are attenuated for colonization of the URT mucosa, and that a capsule mutant is competent to disseminate from the URT to the liver and spleen at wild type levels, possibly involving the NALT and lymphatic system, as has been proposed for *B. pseudomallei* spread by others [7]. Only through studying the capsule mutant in a lung-specific model system have we identified a dissemination defect for this mutant, consistent with a dominant role for capsular polysaccharide as a defense to innate immunity, thereby facilitating disseminated disease. Thus, the study of the role of virulence determinants in respiratory melioidosis may give different phenotypes dependent on whether disease is mediated by URT infection (i.n.) or systemic disease progression (IMIT).

Type 3 Secretion has been characterized as an important *B. pseudomallei* virulence determinant in hamster and murine systemic disease models as well as a murine intranasal model system [18],[25]. *B. pseudomallei* possesses three T3SS clusters in its genome [31], [32], [33], of which only cluster three was found to be important for mammalian virulence, with a calculated attenuation of 10^2.8^ fold in a systemic hamster intraperitoneal model [18]. Given that our IMIT model revealed a reduced importance for the role of capsule in *B. pseudomallei* pulmonary pathogenesis, we investigated whether T3SS3 is an important *B. pseudomallei* virulence determinant in the lung, or whether it too is required preferentially for systemic infection rather than initial lung colonization. Interestingly, we found that a T3SS3 translocation defective mutant was attenuated 10^2.5^ fold, similar to the 10^2.8^ fold attenuation reported in a systemic model. Thus, unlike the capsule mutant which is critical for systemic, but not respiratory, disease, T3SS3 is required ubiquitously for both systemic and respiratory disease. It is understood that a critical phenotype associated with the T3SS3 locus is mediating the ability of *B. pseudomallei* to rapidly escape from the phagosome of professional phagocytes [34], [35]. Thus, T3SS3 mutants exhibit growth defects in intracellular niches associated with delayed vacuolar escape, suggesting that the decreased fitness which we have observed for the T3SS3 mutant in the lung is associated with reduced fitness in the intracellular environment. Our data suggests that *B. pseudomallei* inhabits intracellular niches, not only in the lung, but also in other tissues, which might explain why a similar degree of attenuation is observed for the T3SS3 mutant in both systemic and respiratory disease models. We are therefore interested to identify how specific effector proteins delivered by the T3SS3 apparatus participate in mediating vacuolar escape and increase the fitness of *B. pseudomallei* in the lung.

In summary, we have demonstrated that respiratory melioidosis in the murine model may be associated with severe upper respiratory inflammation which directly drives host morbidity. We have further demonstrated that simple approaches facilitating a lung-specific disease progression allow for abrogation of URT infection, and therefore allow mice to act as much better surrogates for human melioidosis, minimizing the role of URT-based morbidity and CNS involvement. This approach has profound impact both in translational studies as well as basic science investigations. In the case of the former, full disease progression in the mouse will allow an investigation of the efficacy of pre- and post-exposure prophylaxis to protect against an advanced septicemic disease state. With regards to basic science investigations, we have successfully used the IMIT model to meet prediction of the role of capsular polysaccharide in facilitating dissemination of *B. pseudomallei* from the lung, whereas our former intranasal model system did not allow us to draw these predicted conclusions. The IMIT model has also revealed a critical role for the T3SS3 in facilitating respiratory melioidosis.
